# Integrated analysis of the gut microbiome and metabolome in a mouse model of inflammation-induced colorectal tumors

**DOI:** 10.3389/fmicb.2022.1082835

**Published:** 2023-01-13

**Authors:** Yuntian Hong, Baoxiang Chen, Xiang Zhai, Qun Qian, Rui Gui, Congqing Jiang

**Affiliations:** ^1^Department of Colorectal and Anal Surgery, Zhongnan Hospital of Wuhan University, Wuhan, China; ^2^Clinical Center of Intestinal and Colorectal Diseases of Hubei Province, Wuhan, China; ^3^Key Laboratory of Intestinal and Colorectal Diseases of Hubei Province, Wuhan, China; ^4^Department of Infectious Diseases, Southwest Hospital, Third Military Medical University (Army Medical University), Chongqing, China

**Keywords:** colorectal tumor, AOM, DSS, microbiota, metabolite, analysis

## Abstract

Colorectal cancer (CRC) is a common malignancy worldwide, and the gut microbiota and metabolites play an important role in its initiation and progression. In this study, we constructed a mouse model of inflammation-induced colorectal tumors, with fixed doses of azoxymethane/dextran sulfate sodium (AOM/DSS). We found that colorectal tumors only formed in some mice treated with certain concentrations of AOM/DSS (tumor group), whereas other mice did not develop tumors (non-tumor group). 16S rDNA amplicon sequencing and liquid chromatography-mass spectrometry (LC-MS)/MS analyses were performed to investigate the microbes and metabolites in the fecal samples. As a result, 1189 operational taxonomic units (OTUs) were obtained from the fecal samples, and the non-tumor group had a relatively higher OTU richness and diversity. Moreover, 53 different microbes were identified at the phylum and genus levels, including *Proteobacteria*, *Cyanobacteria*, and *Prevotella*. Furthermore, four bacterial taxa were obviously enriched in the non-tumor group, according to linear discriminant analysis scores (log_10_) > 4. The untargeted metabolomics analysis revealed significant differences between the fecal samples and metabolic phenotypes. Further, the heatmaps and volcano plots revealed 53 and 19 dysregulated metabolites between the groups, in positive and negative ion modes, respectively. Styrene degradation and amino sugar-nucleotide sugar metabolism pathways were significantly different in positive and negative ion modes, respectively. Moreover, a correlation analysis between the metabolome and microbiome was further conducted, which revealed the key microbiota and metabolites. In conclusion, we successfully established a tumor model using a certain dose of AOM/DSS and identified the differential intestinal microbiota and characteristic metabolites that might modulate tumorigenesis, thereby providing new concepts for the prevention and treatment of CRC.

## Introduction

Colorectal cancer (CRC) is one of the most common malignancies and a major cause of cancer-related deaths worldwide (Siegel et al., 2020; Sung et al., 2021). In the past 20 years, CRC incidence and mortality have gradually increased, and this disease has tended to affect younger people, especially in China, Japan, and other eastern countries (Dekker et al., 2019; Siegel et al., 2020; Akimoto et al., 2021). To some extent, this could be related to the westernization of diets and lifestyles. Western diets rich in red meat, processed meat, sugar, and refined carbohydrates can increase the risk of colitis-related tumors by changing the intestinal microenvironment, damaging intestinal DNA, and inducing inflammation (Vernia et al., 2021; Arima et al., 2022). However, some intestinal probiotics and beneficial metabolites can effectively antagonize carcinogenesis (Hradicka et al., 2020; Matson et al., 2021). In this context, the role of intestinal microecology changes in CRC initiation and progression is worthy of further exploration.

The inflammation–cancer transformation tumor model, induced with azoxymethane/dextran sulfate sodium (AOM/DSS), is an effective tool to study the mechanisms underlying colorectal tumorigenesis in an inflammatory environment. This animal model, established based on a combination of a mutagen and inflammatory agent, can simulate the entire process of mucosa inflammation-associated tumor formation (Neufert et al., 2007; Angelou et al., 2018). The induced neoplasms in this model mostly occur in the distal colon and first appear in the form of polyps, similar to CRC establishment in humans (Snider et al., 2016). Hence, it can reflect the progression from colitis to carcinoma in humans. However, with respect to AOM/DSS-induced tumorigenesis in mice, we found that with a certain dose, colorectal tumors are successfully induced in some animals, whereas no neoplasm-like changes occur in the others. We speculated that the intestinal microecology of those mice without tumor lesions might have a preventative effect on AOM/DSS-induced carcinogenesis. Therefore, in the current study, 16S rDNA amplicon sequencing and liquid chromatography-mass spectrometry (LC-MS)/MS analyses were used to explore the intestinal flora and metabolites of mice with or without tumors after AOM-DSS treatment, which might help us to further understand the initiation and development of enteritis-related CRC and provide new ideas for the prevention and treatment of CRC from the perspective of the intestinal microecology.

## Materials and methods

### Animals and treatment

Animal experimentation was approved by the Animal Committee of the Chinese Academy of Sciences Institutional Laboratory [WIVA042020003]. In total, 70 female C57BL/6 mice (6-weeks-old, 20–24 g) were used in this study. AOM was purchased from Sigma-Aldrich (No. A5486, USA), and DSS was purchased from MP Biomedicals (No. 160110, CA). The animal experiments were conducted in two stages.

**FIGURE 1:**
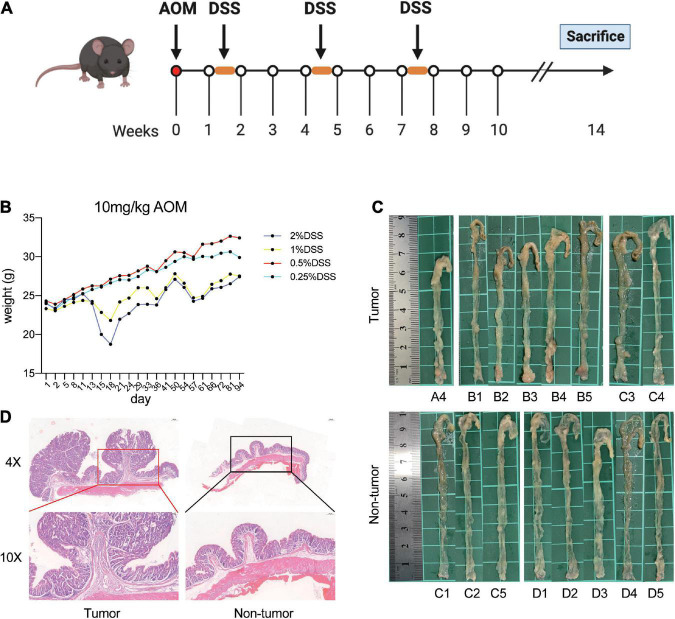
Establishment of AOM/DSS-induced mice models. **(A)** Flow chart of the mice treated with AOM/DSS. **(B)** Weight of mice in each group during AOM/DSS treatment. **(C)** Macroscopic view of colon. **(D)** Representative hematoxylin and eosin stain of the distal colon with tumor and no tumor.

In the first stage, 35 mice were randomly divided into seven groups (*n* = 5) and treated with AOM/DSS at different concentrations as follows: Group A, 10 mg/kg AOM, 2% DSS; Group B, 10 mg/kg AOM, 1% DSS; Group C, 10 mg/kg AOM, 0.5% DSS; Group D, 10 mg/kg AOM, 0.25% DSS; Group E, 7.69 (10/1.3) mg/kg AOM, 2% DSS; Group F, 5.92 (10/1.3^2^) mg/kg AOM, 2% DSS; Group G, 4.55 (10/1.3^3^) mg/kg AOM, 2% DSS. The mice were intraperitoneally injected with AOM on the first day. Then, 1 week later, the mice were treated with DSS solution for 1 week, followed by 2 weeks of normal drinking water, for three cycles. All mice were euthanized until 14 weeks. The animal modeling process is shown in Figure 1A.

In the second stage, 35 mice were randomly divided into an experimental group (*n* = 30) and control group (*n* = 5). Here, we hypothesized that DSS at a certain dose might result in tumors in 50% of mice, without tumors in the other 50% of mice. The expected dose of DSS was calculated as 0.5359% according to the method in a previous study (Sanchez et al., 2018). Mice in the experimental group were treated with 10 mg/kg AOM and 0.5359% DSS in drinking water, based on the aforementioned procedure. Meanwhile, mice in the control group were maintained under standard conditions for 14 weeks. Finally, the colon tissues and fecal samples of all mice were collected for further investigation.

### 16S rDNA amplicon sequencing

The fecal genomic DNA was extracted using a Stool DNA Kit (Qiagen, Germany) according to the manufacturer’s experimental steps. After determining the DNA integrity and concentration, the qualifying DNA samples were used for amplification. Specific primers were designed for the 16S rRNA V3–V4 region (F: 5′-CCTACGGGAGGCAGCAG-3′; R: 5′-GGACTACHVGGGTATCTAAT-3′). PCR amplification was performed using High-Fidelity PCR Master Mix with GC Buffer (New England Biolabs, USA). Then, the PCR products were separated using 2% agarose gel electrophoresis and magnetic beads and purified with a Gel Extraction Kit (Qiagen, Germany). The amplicon libraries were constructed using a TruSeq^®^ DNA PCR-Free Sample Preparation Kit (Illumina, USA), then quantified with a Qubit and qPCR, and finally sequenced on a NovaSeq6000 (Illumina, USA) platform. The effective tags were analyzed and obtained with the assistance of Novogene Biotechnology (Guangzhou, China). Operational taxonomic units (OTUs) were clustered based on tags of more than 97% identity using the Uparse v7.0.1001 method. Further analyses, including alpha and beta diversity, were subsequently performed.

### Untargeted metabolomics analysis

Untargeted metabolomics were investigated via LC-MS/MS analyses. Fecal samples (100 mg) were placed in Eppendorf tubes and quickly treated with liquid nitrogen. Then, the samples were resuspended well with 80% methanol and 0.1% formic acid. After incubation for 5 min in an ice bath, the mixture was centrifuged at 15,000 × *g* and 4°C for 20 min. The supernatants were transferred and diluted with LC-MS grade water with methanol at a final concentration of 53%. Following another centrifugation step at 15,000 × *g* and 4°C for 15 min, the resulting supernatants were collected for subsequent experiments.

LC-MS/MS analyses were performed using the Vanquish UHPLC system and Orbitrap Q Exactive™HF-X mass spectrometer (Thermo Fisher Scientific, Germany) provided by Novogene (Beijing, China). The samples were injected into a Hypesil Gold column (2.1 mm × 100 mm, 1.9 μm), and the flow rate was 0.2 ml/min. Eluent A was 0.1% formic acid in water, and eluent B was methanol for the positive polarity mode, whereas for the negative polarity mode, eluent A was 5 mmol/L ammonium acetate in water, pH 9.0, and eluent B was methanol. The solvent gradient was set as follows: 1.5 min, 2% B; 12 min, 2–100% B; 14 min, 100% B; 14.1 min, 98% B; 17 min, 2% B. A QExactiveTMHF-X mass spectrometer was used with the source conditions as follows: sheath gas flow rate of 40 Arb, aux gas flow rate of 10 Arb, spray voltage of 3.2 kV, capillary temperature of 320°C. The raw data files were generated based on UHPLC-MS/MS and processed using Compound Discoverer 3.1 (Thermo Fisher, USA).

### Statistical analysis

Qiime software (Version 1.9.1) was used to calculate Observed-OTU, Chao1, Shannon, and Simpson indices. Differences in alpha diversity indices among groups were analyzed based on the rarefaction curve and rank abundance curve. A Wilcox test was used for alpha diversity and beta diversity analyses. ANOSIM analysis was performed to test for differences in the microbial communities among groups. Raw data of LC-MS/MS were analyzed using Compound Discoverer 3.1. The differences in metabolic patterns among different groups were revealed based on partial least squares discrimination analysis (PLS-DA). To study phenotypic changes that might be caused by changes in the host microbial community structure, correlation analyses between the microbiome and metabolome were performed based on Pearson’s correlation analysis, correlation network diagram analysis, and correlation Sankey diagram analysis. *P* < 0.05 was considered statistically significant.

## Results

### Generation of AOM/DSS-induced tumor mouse models

First, the mice were treated with AOM/DSS at different concentrations. After the initiation of tumorigenesis for 14 weeks, the mice were euthanized and investigated. We observed that mice in the group administered 1 and 2% DSS lost significantly more weight than those in the other two groups (Figure 1B). Moreover, the nodular tumors were macroscopically visible in the distal colon of mice in different groups (Figure 1C). In addition, the mice had obvious tumors in group A and B, whereas group D had no tumors (Table 1 and Figures 1C, D). However, the mice treated with 2% DSS and different concentrations of AOM had poor survival outcomes (Table 1). Therefore, we selected DSS as a variable factor to establish the target mouse models.

**TABLE 1:** Incidence of tumor in mice treated with different doses of AOM/DSS.

Group (*n* = 5)	Application		Survival	With tumor	Incidence (%)
	AOM (mg/kg)	DSS (%)			
A	10	2	1	1	100
B	10	1	5	5	100
C	10	0.5	5	2	40
D	10	0.25	5	0	0
E	7.69	2	2	2	100
F	5.92	2	3	2	66.67
G	4.55	2	2	1	50

Here, we hypothesized that DSS at a specific dose would cause 50% of mice in a test population to develop tumors, and the theoretical dose was 0.5359% based on the results of groups A–D. Next, the mice in the experimental group (*n* = 30) were treated with 10 mg/kg AOM and 0.5359% DSS, and a negative control group of mice (*n* = 5) was also used in parallel. Fourteen weeks later, the mice were euthanized and investigated. As a result, 12 mice had tumors and 18 mice had no tumors in the experimental group. Then, we randomly selected the nine mice with tumors (tumor group), nine mice with no tumors (non-tumor group), and five control mice (control group) for further experiments and analysis.

### Alterations to the gut microbiomes in different groups

**FIGURE 2:**
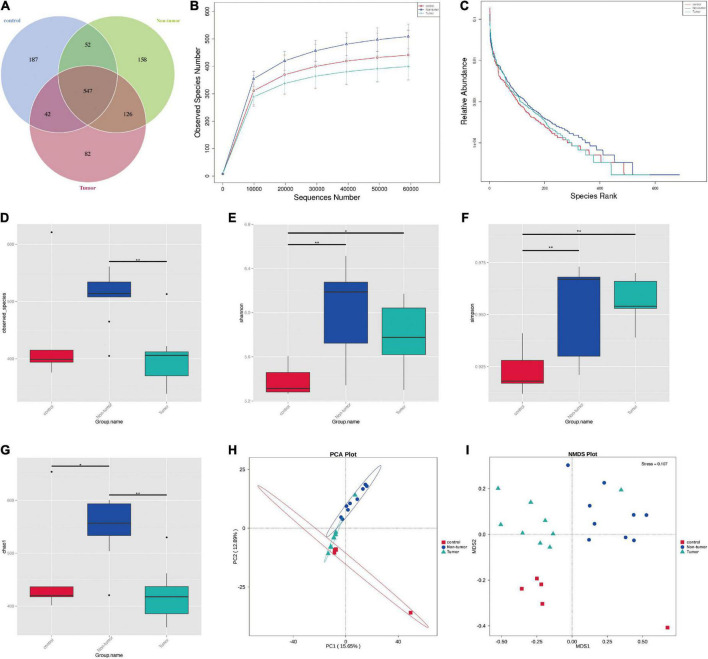
The diversity of the microbial communities in three groups. **(A)** Venn diagram shows the compositions of OTUs. **(B)** Rarefaction curve. **(C)** Rank abundance curve. **(D–G)** Alpha diversity index analysis (ACE, Shannon, Simpson, and Chao1). **(H)** Principal component analysis; **(I)** NMDS analysis. *p < 0.05, **p < 0.01.

To explore whether tumorigenesis is related to the gut microbiome, 16S rRNA sequencing was performed to identify gut microbiota profiles. In total, 1,189 OTUs were obtained among the three groups, comprising 828 in the control group, 797 in the tumor group, and 883 in the non-tumor group (Figure 2A). Moreover, a relative increase in bacterial richness was found in the non-tumor group, as revealed based on the rarefaction curve, compared with that in the other two groups (Figure 2B). In addition, the rank abundance curve yielded similar results (Figure 2C). To investigate bacterial diversity, we analyzed the alpha diversity indices and observed that there were statistically significant differences in the observed species, Shannon, Simpson, and Chao1 indices among different groups (Figures 2D–G). The principal component analysis showed that there were three separations of gut microbiota distributions among the three groups (Figure 2H). Non-metric multi-dimensional scaling analysis also revealed different distributions of microbial communities among the three groups (Figure 2I).

### Identification of differential bacteria among different groups

**FIGURE 3:**
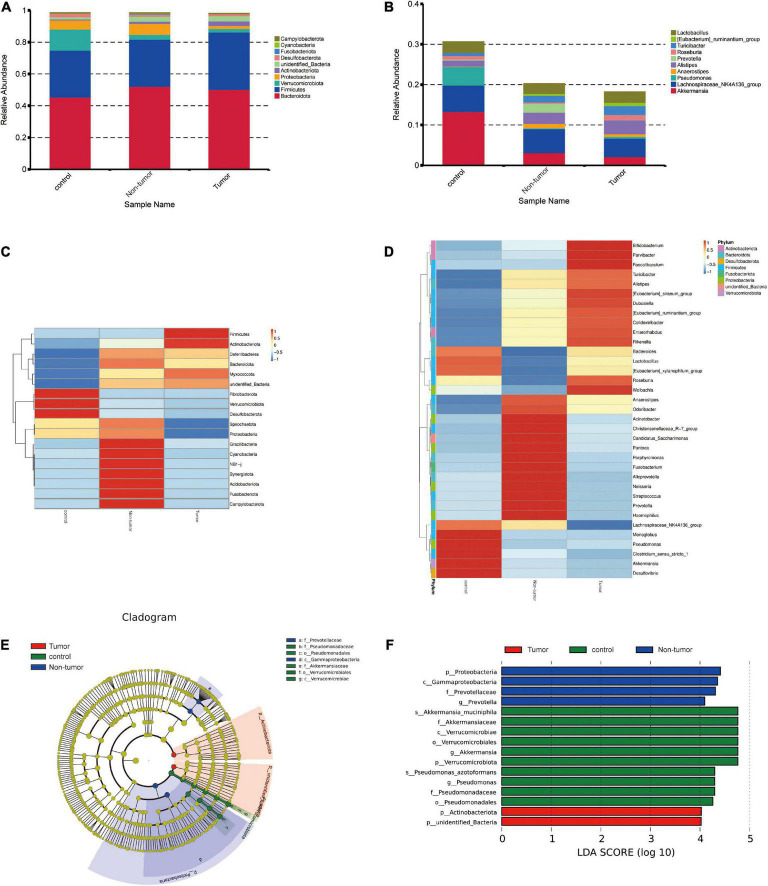
Identification of the differential bacteria from the three groups. **(A,B)** Component proportion of bacteria at the phylum **(A)** and genus **(B)** level in different groups. **(C,D)** Heat maps to identify different fecal microbiota at the phylum **(C)** and genus **(D)** level in the different groups. **(E)** The cladogram to show specific differential bacteria in the three groups. **(F)** LEfSe indicating the different bacterial taxa.

The gut microbial community structures at the phylum and genus levels in the three groups were analyzed, and the top 10 differences are presented in Figures 3A, B. The differences in the microbial distribution among the groups were determined through ANOSIM analysis (Supplementary Figures 1A–C). Next, the differential component proportions of microbes in each group were revealed based on the heatmaps (Figures 3C, D). In addition, at the phylum level, we observed that the Cyanobacteria, Proteobacteria, and Fusobacteriota were significantly enriched in the non-tumor group compared to abundances in the tumor groups, and Verrucomicrobiota was more abundant in controls (Supplementary Figures 1D–F). At the genus level, *Prevotella*, *Alloprevotella*, *Neisseria*, and *Akkermansia* exhibited marked differences among the groups (Supplementary Figures 1G–I).

To further determine the specific gut microflora associated with colorectal tumorigenesis, linear discriminant analysis effect size was performed among the three groups. The branching maps containing six levels, from phylum to species, reveled the signature microbiota. We found that the family Prevotellaceae and class Gammaproteobacteria may have a great effect in the non-tumor group, whereas the families Pseudomonadaceae and Akkermansiaceae, orders Pseudomonadales and Verrucomicrobiales, and class Verrucomicrobiae might play important roles in the control group (Figure 3E). Moreover, based on linear discriminant analysis scores (log_10_) > 4, the histogram showed that four bacterial taxa, including Proteobacteria, Gammaproteobacteria, Prevotellaceae, and Prevotella, were enriched in the non-tumor group, two bacterial taxa were enriched in the tumor group, and 10 bacterial taxa were enriched in the control group (Figure 3F).

### Changes in fecal metabolites among different groups

**FIGURE 4:**
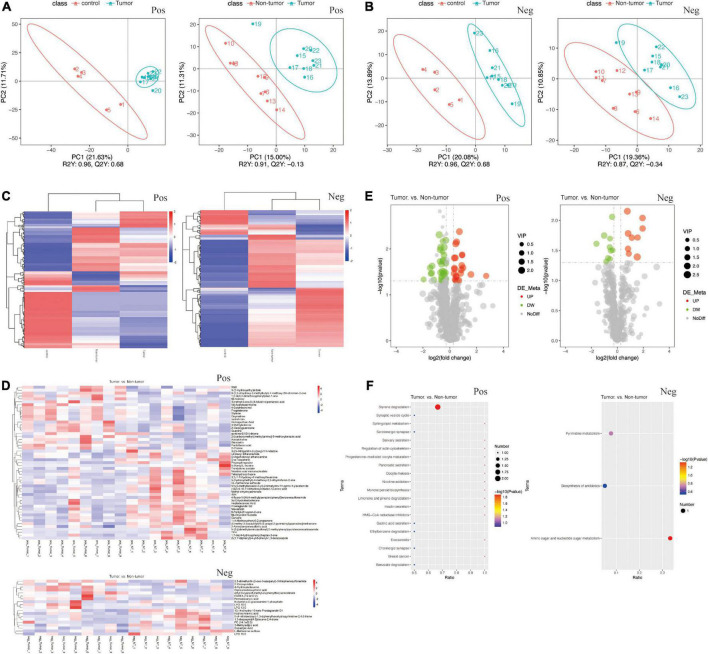
Changes of the fecal metabolites in the groups. **(A,B)** PLS-DA of fecal samples between tumor group and control group **(A)**, between tumor group and non-tumor group **(B)**, by the positive and negative ion methods. **(C)** Heat maps of different fecal metabolites among three groups. **(D)** Heat maps of different metabolites in each fecal samples between tumor group and non-tumor group. **(E)** Volcano Plots indicating the variation of fecal metabolites between the two groups. **(F)** KEGG pathway analysis of metabolism between the two groups.

To identify the signature metabolites from fecal samples among the groups, we performed untargeted LC-MS/MS-based metabolomics. The PLS-DA showed significant differences between the fecal samples and metabolic phenotypes of different groups in both positive and negative ion modes (Figures 4A, B). In total, 1,112 and 554 metabolites were found to be changed in the tumor group, non-tumor group, and control group, in positive and negative ion modes, respectively (Supplementary Table 1). The differences in metabolites among the three groups are shown in Figure 4C. Further, we focused on the differences between the tumor group and non-tumor group. The heatmaps revealed the metabolite differences across each sample within the two groups (Figure 4D). The volcano plots also showed the significant upregulated or downregulated metabolites in the tumor group compared with levels in the non-tumor group (Figure 4E). Briefly, in positive ion mode, levels of 31 and 22 fecal metabolites were up- and downregulated, respectively, in the non-tumor group, with statistically significant differences compared to those in the tumor group. Meanwhile, in negative ion mode, levels of 10 and nine fecal metabolites were significantly up and downregulated, respectively, in the non-tumor group, with statistically significant differences compared to those in the tumor group. The structures of these metabolites were diverse, with many of the metabolites being either directly generated or modulated by the gut bacteria, including homogentisic acid, 3-methyladenine, and 2’-deoxyguanosine (downregulated in positive ion mode); nicotinic acid mononucleotide, N-acetyl-L-leucine, and linoleoyl ethanolamide (upregulated in positive ion mode); glycoursodeoxycholic acid, 2’-deoxyuridine, and pentadecanoic acid (downregulated in negative ion mode); and hydrocinnamic acid, oxoadipic acid, and 3-methyladipic acid (upregulated in negative ion mode). Moreover, Kyoto Encyclopedia of Genes and Genomes (KEGG) pathway analysis was used to identify the enriched pathways associated with differential metabolites in the two groups, and the top 20 most enriched pathways are listed in Figure 4F. Among them, styrene degradation and amino sugar-nucleotide sugar metabolism, were significantly altered in positive and negative ion modes, respectively.

### Correlation analysis between the gut microbiota and metabolites

**FIGURE 5:**
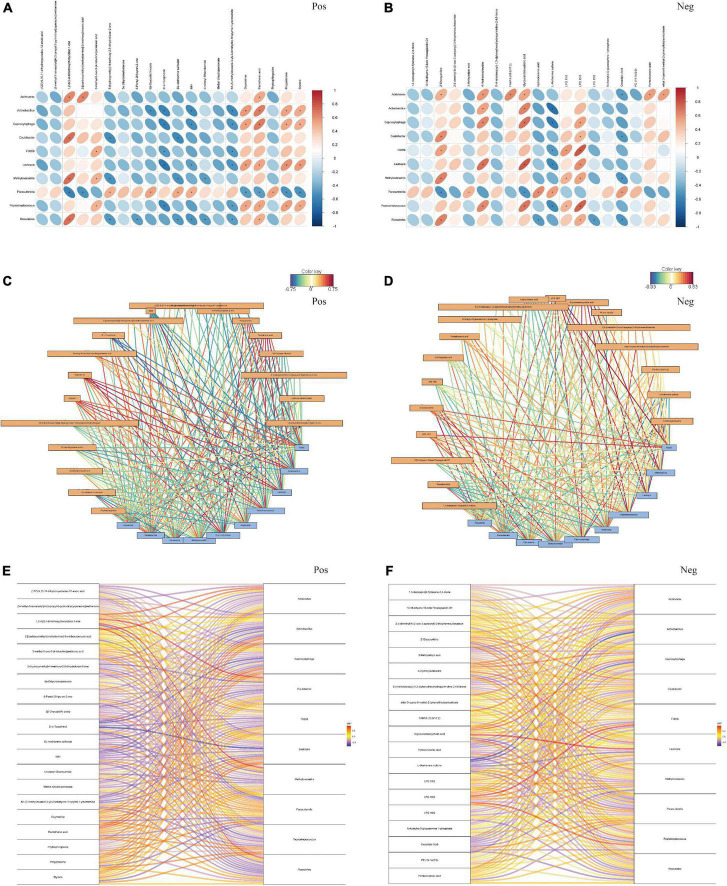
The association analysis between top 10 differential fecal microbiota at genus level and top 20 differential metabolites. **(A,B)** Pearson correlation coefficient analysis. **(C,D)** Correlation network diagram analysis. **(E,F)** Correlation Sankey diagram analysis, by the positive and negative ion methods, separately. Red represented that the microbiota was positively correlated with metabolites, and bule represented that the microbiota was negatively correlated with metabolites. *P < 0.05.

To investigate the association between differential microbiota and metabolites in fecal samples, we conducted correlation analysis based on top 10 different bacteria at the genus level and top 20 different metabolites between the tumor group and non-tumor group. As shown in Figures 5A, B, the correlation heatmaps revealed the association between metabolites and microbiota, based on Pearson correlation coefficient analysis, in positive and negative ion methods. To further reveal the key bacterial flora and metabolites, we generated correlation network diagrams and observed that the connections were multiple and consanguineous (Figures 5C, D). Moreover, the correlation Sankey chart analysis also visually demonstrated the association between the gut microbiota and metabolites (Figures 5E, F). Notably, in positive ion mode, we observed that D-α-tocopherol was significantly negatively correlated with most microbiota, including *Actinobacillus*, *Capnocytophaga*, F0058, *Lautropia*, and *Peptostreptococcus*. Similarly, N1-(5-methylisoxazol-3-yl)-2-tetrahydro-1H-pyrrol-1-ylacetamide also exhibited negative correlations with most microbes. Meanwhile, 1,2-di(3,4-dimethoxyphenyl)diaz-1-ene, 3-methyl-5-oxo-5-(4-toluidino)pentanoic acid, oxymatrine, pantothenic acid, progesterone, and styrene showed positive correlations with the vast majority of microbe–metabolite pairs. In negative ion mode, the results showed highly negative associations for several microbe–metabolite pairs, such as *Actinobacillus*/L-methionine sulfone, *Capnocytophaga*/L-methionine sulfone, and *Parasutterella*/2’-deoxyuridine. However, *Lautropia*/glycoursodeoxycholic acid, *Capnocytophaga*/glycoursodeoxycholic acid, *Lautropia*/4-hydroxyisoleucine, and F0058/LPG 15:0, among others exhibited opposite relationships. Taken together, these results revealed significant correlations with respect to key microbe–metabolite pairs in the tumor and non-tumor groups, suggesting their potential roles in modulating tumorigenesis.

## Discussion

The AOM/DSS-induced mouse model is a common experimental tumor model to develop colitis-associated colon cancer. Specifically, it can mimic the non-hereditary features of CRC in terms of the normal epithelium/adenoma/carcinoma progression (Neufert et al., 2007; Dekker et al., 2019). Further, it is an essential tool to investigate the underlying mechanisms of CRC initiation and progression, but it is also a valuable and effective model for the evaluation of novel therapeutic options. For example, Wei et al. elucidated the role and molecular mechanism of NDRG2 in tumor development using AOM/DSS mice (Wei et al., 2020). Moreover, Gobert revealed the protective function of spermine oxidase in colon inflammation and tumorigenesis (Gobert et al., 2022).

In previous studies, researchers have devoted time to finding the optimal conditions of AOM and DSS utilization to induce tumor development, including the doses of AOM and/or DSS (Bissahoyo et al., 2005; Suzuki et al., 2005; Neufert et al., 2007; Angelou et al., 2018). Interestingly, we observed that when mice were administrated various concentrations of AOM/DSS, different tumor burdens were noted. Compared to those in the 5 mg/kg AOM group, the percentage of tumor-bearing mice, tumor multiplicity, and size were significantly increased in the 10 mg/kg AOM group, whereas 20 mg/kg AOM resulted in acute toxicity (Bissahoyo et al., 2005). Moreover, in another study, Suzuki administered 10 mg/kg AOM to the mice, followed by DSS solution at levels of 0.1, 0.25, 0.5, 1, and 2%. The incidences of neoplasms were 0, 0, 20, 100, and 100% for each group, respectively (Suzuki et al., 2005). To some extent, the tumor-promoting ability of AOM/DSS might thus be dose-dependent. Therefore, we hypothesize that there is a certain dose of these chemical agents that could lead to a 50% possibility of tumor development.

In our study, we generated the mouse models with multiple combinations of AOM and DSS doses, as presented in Table 1. As a result, we found increasing incidences of tumors in the mice treated with DSS, from 0.25 to 2% (the dose of AOM was 10 mg/kg). Among these concentrations, 1 and 2% DSS resulted in a tumor incidence of 100%, whereas 0.5 and 0.25% DSS induced lower incidences, specifically less than 50%. However, 2% DSS treatment led to poor survival outcomes in the groups. Thus, we set the AOM dose at 10 mg/kg and the targeted dose of DSS at 0.5359% for the following animal experiment (*n* = 30), which was thought to be associated with a theoretical 50% probability of tumor initiation. Finally, in the experimental group, 12 mice developed tumors and 18 mice had no tumors. We next sought to determine what factors contribute to this phenomenon.

With the continuous progress of high-throughput sequencing and bioinformatics, studies on the human intestinal flora have been further developed. Numerous studies have indicated that genetic and environmental factors play important roles in carcinogenesis (Song et al., 2015; Yang et al., 2019). CRC occurs directly in the gut and is therefore closely related to changes in the intestinal microecology. Accumulating evidence demonstrates that dysbiosis of the intestinal flora could modulate the progression, development, and treatment of CRC (Louis et al., 2014; Feng et al., 2015; Fong et al., 2020; Huang et al., 2020, 2022). For example, a recent study showed that CRC patients have gut microbiome imbalances, which were characterized by an increase in the abundance of cancer-related bacteria, such as pks + *Escherichia coli*, enterotoxigenic *Bacteroides fragilis*, and *Fusobacterium nucleatum*, whereas the abundance of beneficial bacteria such as *Roseburia*, *Clostridium*, and *Bifidobacterium* were found to be decreased (Janney et al., 2020). Similarly, in the current study, the abundances of *Colidextribacter* and *Bacteroides* were increased in the tumor group, suggesting that these harmful bacteria might participate in the process of colorectal tumorigenesis. Interestingly, although the abundance of the beneficial bacteria *Clostridium* increased in the tumor group, other probiotics commonly believed to play a role in CRC, such as *Bifidobacterium* and *Roseburia*, did not show a decrease in abundance in the tumor group, indicating that changes in the composition of gut microbes during inflammation-mediated colorectal tumorigenesis might be different from those occurring with conventional CRC. These so-called “abnormal” intestinal flora changes deserve further study and discussion, in the context of the AOM/DSS-mediated inflammation tumor animal model. Moreover, at the phylum level, we observed that a variety of microbes, such as Proteobacteria and Cyanobacteria, were significantly increased in the non-tumor group compared to the abundance in the tumor group. Proteobacteria, as a source of natural products, provides unappreciated potential to discover and develop novel bioactive molecules with antibiotic and anticancer effects (Buijs et al., 2019). Cyanobacteria and its metabolites also have favorable potential as anticancer drugs (Mondal et al., 2020).

Metabolomics can clearly reflect the functional changes in the gut microbiota under specific conditions through the detection of metabolites, which might provide clues to reveal the relationship between the gut microbiota and the occurrence and development of diseases (Han et al., 2021; Krautkramer et al., 2021; Bauermeister et al., 2022). Our metabolomic analysis showed that 72 metabolites in the non-tumor group were significantly changed, compared with levels in the tumor group. Among them, tetrahydrocortisone, O-arachidonoyl ethanolamine, and D-α-tocopherol were enriched in the non-tumor group. These metabolites and their analogs have some anti-inflammatory properties. For example, as an endogenous cannabinoid, O-arachidonoyl ethanolamine has been proven to be an endogenous inhibitor of cytochrome P450 cyclooxygenase, with anti-inflammatory effects (Carnevale et al., 2018). Moreover, tetrahydrocortisone is a metabolic product of hydrocortisone, and its enrichment indicates that the glucocorticoid anti-inflammatory pathway might be active (Wang et al., 2018; Sagmeister et al., 2019). D-α-Tocopherol can play an anti-inflammatory role by reducing the release of proinflammatory cytokines (such as interleukin-1 β, interleukin-6, and tumor necrosis factor α) and chemokines (such as interleukin-8) and reducing the adhesion of monocytes to the endothelium. The KEGG pathway analysis showed that some pathways, such as styrene degradation and amino sugar-nucleotide sugar metabolism, were significantly enriched between the groups. Interestingly, environmental nanoparticles, especially polystyrene nanoparticles, are a potential risk for intestinal injury. It has been reported that PNP exposure can induce cytotoxic and genotoxic effects on cells by inducing oxidative stress related to nuclear damage (Vecchiotti et al., 2021). Homogentisic acid is a metabolite annotated to the styrene degradation pathway, and it has been proven to be cytotoxic for various cell lines (Jurič et al., 2022). However, whether it can participate in CRC is worth further exploring.

It is known that the gut microbiome can regulate metabolic homeostasis, and we further conducted correlation analysis of the gut microbiome and metabolome. Notably, in our findings, D-α-tocopherol, an anti-inflammatory factor enriched in the non-tumor group, was significantly negatively correlated with several microbes, such as *Actinobacillus*, *Capnocytophaga*, and *Lautropia*. A previous study revealed that *Actinobacillus* had the higher degree of centrality across the progression of precancerous lesions of gastric cancer, and *Acinetobacter* might contribute to the occurrence of intraepithelial neoplasia (Liu et al., 2021). *Capnocytophaga*, an oral bacterium, was also found to be highly present in oral squamous cell carcinoma tissues and exert tumor-promoting effects on oral cancer (Zhu et al., 2022). Moreover, Li et al. (2020) revealed that *Lautropia* was enriched in hepatitis patients and might participate in the progression of liver cancer. Therefore, in the future, more experiments should be performed to validate the effect of the identified microbiota and metabolites on CRC progression and treatment.

It should be noted that, whether the changes in the gut microbiota could affect disease development or the occurrence of disease may cause an imbalance in the intestinal flora, as well as the mechanism underlying such phenotypes, need to be further elucidated. Moreover, although some differential microbiota and metabolites were identified in the animal models, their antitumor effects in animal models and in humans have not been further demonstrated. Despite this, our study investigated the intestinal microecology of colorectal tumors using an AOM/DSS mouse model, with a specific concentration used for treatment. We demonstrated the differentially abundant microbiota and metabolites in the gut and identified the potential key relationships between them. These findings might provide guidance to elucidate the mechanism underlying the pathogenesis of inflammation-mediated colorectal tumors.

## Conclusion

In this study, we successfully generated an AOM/DSS mouse model, based on a certain dose that could influence the development of CRC. Using this model, 16S sequencing and LC-MS/MS analyses were performed to identify and explore the differential gut microbiota and metabolites that might be associated with tumorigenesis. This could ultimately provide a new direction for the prevention and treatment of CRC.

## Data availability statement

The raw data supporting the conclusions of this article will be made available by the authors, without undue reservation.

## Ethics statement

This animal study was reviewed and approved by Animal Committee of Chinese Academy of Sciences Institutional Laboratory (WIVA042020003).

## Author contributions

CQJ and RG conceived and designed experiments. YTH, RG, and BXC performed the experiments. YTH, BXC, XZ, and QQ interpreted the results of experiments. YTH wrote the manuscript. All authors reviewed and approved the final manuscript.
