# Supplementary material for: Integrated analysis of the gut microbiome and metabolome in a mouse model of inflammation-induced colorectal tumors
Source: Front Microbiol. 2023 Jan 13;13:1082835. doi: 10.3389/fmicb.2022.1082835 (PMC9880073; doi:10.3389/fmicb.2022.1082835)
Supplement: Supplementary file 1 [file Data_Sheet_1.DOCX]

**Additional file**

**
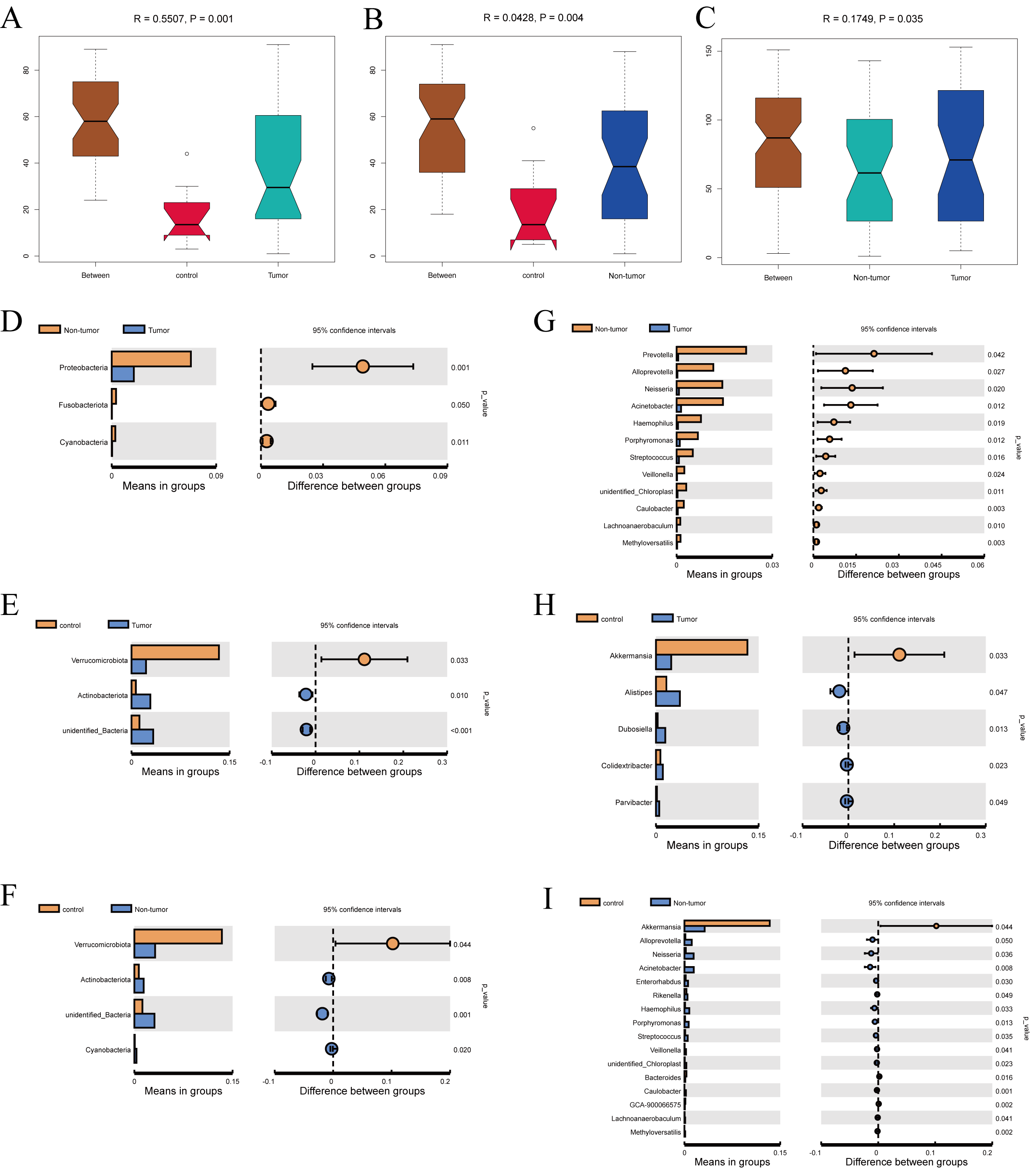
**

**FIGURE S1** | Differences of the fecal microbes in the groups. **(A-C)** Anosim analysis for the fecal samples between each two groups; **(D-F)** T-test for differences of microbiota between groups at phylum level; **(G-I)** T-test for differences of microbiota between groups at genus level.

**Table S1** Differences of fecal metabolites among groups.

| **Compared Samples** | **Num. of Total Ident.** | **Num. of Total Sig.** | **Num. of Sig.Up** | **Num. of Sig.down** |
| --- | --- | --- | --- | --- |
| Tumor.vs.Non-tumor_pos | 1112 | 53 | 22 | 31 |
| Non-tumor.vs.control_pos | 1112 | 187 | 98 | 89 |
| Tumor.vs.control_pos | 1112 | 191 | 75 | 116 |
| Tumor.vs. Non-tumor_neg | 554 | 19 | 9 | 10 |
| Non-tumor.vs.control_neg | 554 | 115 | 99 | 16 |
| Tumor.vs.control_neg | 554 | 98 | 75 | 23 |
